# A new member in the EJNMMI family: the European Journal of Hybrid Imaging

**DOI:** 10.1186/s41824-017-0010-2

**Published:** 2017-10-12

**Authors:** Arturo Chiti

**Affiliations:** Humanitas University and Humanitas Research Hospital, Milano, Italy

The term “hybrid” in biology refers to an offspring resulting from cross-breeding. Sometimes these hybrids are sterile. This is not the case in imaging, where hybrid “creatures” are giving birth to several “fertile” studies, clinical applications and educational programs. A prominent component of these offspring is represented by publications and this is why EANM and Springer-Nature decided to launch a new imaging journal: the EJHI.

In imaging, the adjective hybrid is used when two technologies are used at the same time, or within a short time interval, to study the same patient. The rationale of this approach relies in the possibilities of taking advantage of the combined used of the single techniques. In other words, as it was written years ago: 1 + 1 is more than 2.

The medical community is also using the term multimodality imaging, when referring to procedures implying the use of different modalities, not necessarily at the same time. Typical examples are: PET and CT, or SPECT and CT, or PET and MR.

Hybrid imaging represents the great majority of the daily workflow in a number of imaging departments across the world at it is used in several research applications.

Industries put a lot of efforts in developing hardware and software tools able to make hybrid scanners as effective as single modalities scanners in the daily and research use. How these efforts were successful can be tested by everyone working in the field and can be read in thousands of publications already made with hybrid technologies.

Besides new generation scanners, hybrid imaging relies on radiopharmaceuticals. Without radiopharmaceuticals, we would not have seen hybrid imaging like it is today; their diagnostic use in combination with CT, MR, US and optical probes can exploit all the potential of clinical and preclinical imaging.

The challenge, from a clinical point of view, it is to have these paired modalities used with their full potentials. Still after several years from the introduction of the technology, we often see duplication of procedures just because one of the hybrid components of imaging is under used or under evaluated.

Taking full advantage from the hybrid technology needs a significant effort that can be summarized in few steps.

First of all, we need to favour an increased availability of radiopharmaceuticals: many molecules cannot be used due to regulatory constraints that make clinical trials expensive and difficult to run in many countries. Then, the introduction in clinical practice is also expensive and slow, thus reducing the possibilities for the patients to benefit from the technology. Also, different rules apply in different countries, causing inequality in patients’ access to modern diagnostic procedures within the EU.

As a second step, we need to promote educational programmes dedicated to hybrid imaging. Several scientific societies are offering educational activities aimed at developing the next generation of imagers. Indeed, this is the field in which we see more activities. In particular, EANM’s European School of Multimodality Imaging and Therapy is making a great effort to provide all the competences and skills required in order to correctly use and interpret hybrid imaging. A challenge will be to offer courses at a national level, in order to increase knowledge and confidence in hybrid imaging among the whole medical community.

Another essential action is the introduction of a legal framework that allows nuclear medicine physicians and radiologists to have full access to hybrid imaging, going beyond the obsolete requirement of double certification. Programs should be created to certify hybrid imagers to read all diagnostic modalities employed in hybrid imaging. These programs should focus on the combined use of the different technologies and should be accessible to new specialists and to those who are already practicing imaging with hybrid modalities.

As scientists, we have to generate evidence on the clinical advantages of hybrid imaging, in order to justify the use of the expensive hybrid imaging techniques. This means to design, finance, and promote clinical trials that focus on demonstrating the advantages of using the hybrid technologies to properly address unmet clinical needs.

The *European Journal of Hybrid Imaging* can be seen as one of the facilitators to put in practice the points mentioned above. The journal is a platform to publish papers focusing on the use of hybrid and multimodality imaging in clinical practice, clinical trials, and preclinical research, able to increase the diffusion of scientific reports on hybrid imaging.

We are confident that the EJHI will actively contribute to the creation of a bridge between the different modalities employed in imaging and will facilitate their use by the scientific community.

Together with the other EJNMMI family journals, the EJHI will act to facilitate the development of this evolution of imaging and the diffusion of the most important clinical results.

*Arturo Chiti.*

*Editor in Chief, European Journal of Hybrid Imaging.*

